# Realigning identity: Nurse executives' experiences within a new socio-professional group – A classic grounded theory study

**DOI:** 10.1016/j.ijnsa.2025.100367

**Published:** 2025-06-14

**Authors:** Cora Lunn, Claire O’ Donnell, Sarah MacCurtain, Alice Coffey

**Affiliations:** a School of Nursing and Midwifery, University of Limerick, Limerick, Ireland; b Kemmy Business School, University of Limerick, Limerick, Ireland; c Health Research Institute, University of Limerick, Limerick, Ireland

## Abstract

**Background:**

Nurse executives play a crucial role in adapting to the evolving needs of healthcare communities. Existing research demonstrates the positive impact of nursing leadership practices on workforce retention, job satisfaction and overall well-being. The complexities surrounding role transitioning for nurse executives remains under explored.

**Objective:**

This study aims to deepen the understanding of how nurse executive leaders navigate the intricate process of role transitioning, providing insights into their experiences and challenges.

**Design:**

This study was conducted using Glaser's classic grounded theory.

**Setting:**

This study was carried out in acute hospital settings at seven different sites in the Republic of Ireland.

**Participants:**

Participants were 12 nurse executives who were working in the Republic of Ireland, with additional reflective diary notes gathered from conversations with six international nurse executives.

**Methods:**

Data were collected through unstructured interviews conducted between January 2020 and September 2022. The process of data collection and analysis occurred simultaneously, with the data being analysed based on the principles of classic grounded theory.

**Results:**

This study introduces the theory of Identity Realigning, which describes the leadership development of nurse executives through three stages: identity earning, role transitioning, and self-integrating. Identity Earning involves forming a new identity within a new socio-professional group. Role transitioning is the psychological process of moving from one role to another, encompassing various stages. Self-Integrating refers to the extent of assimilation into the new role and socio-professional group. Factors such as organisational design and resources were identified as contextual conditions that can either facilitate or impede success for nurse executives.

**Conclusion:**

The theory of Identity Realigning provides significant insights for educators, leaders, and policymakers by explaining the intricate process of role transitioning for nurse executives. It establishes a clear link between shifts in professional identity and the processes of role transitioning and integration. This connection underscores the critical importance of developing executive skills and highlights the necessity for tailored professional development strategies for this cohort.

Identity realigning theory; Nurse executive; Identity earning; Role transition; Self-integration

**What is already known**

• Role transitions in healthcare leadership are challenging, with limited research on identity adaptation for nurse executives.
• Existing theories offer limited insights into the specific dynamics faced by nurse executives in socio-professional integration.

Alt-text: Unlabelled box

**What this study adds**

• Introduces the concept of Identity Realigning, emphasising identity earning, role transitioning, and self-integration.
• Highlights the interplay between confidence, courage, and socio-professional relationships as pivotal to professional identity formation.
• Offers practical insights for leadership development, including tailoring onboarding and mentorship programmes.

Alt-text: Unlabelled box

## 1 Introduction

One of the cornerstones of healthcare leadership is undoubtedly the service of nursing leaders to patient care. The World Health Organisation (WHO) and the International Council of Nurses (ICN) have stressed that leadership capacity is essential for all nurses, in particular nurse managers (Duygulu and Kublay 2008; Kantek et al., 2023). Nurse executive leadership styles and behaviours can strongly impact the nursing workforce, their work environment and well-being (Niinihuhta and Häggman-Laitila 2022). Healthcare organisations demand strong nursing leadership at executive level, and these roles require distinctive executive competencies (Lee et al. 2019; Leclerc et al. 2021).

Nurse executives play a crucial role in aligning organisational goals, values, and philosophy within healthcare settings and translate the corporate perspective to the nursing workforce (Clark 2012; González García et al. 2022). They act as integral partners in decision-making processes and bear responsibility for ensuring the provision of safe, high-quality care (Schoville et al. 2022). Sundean et al. (2022) highlight that senior nurse leaders provide a unique understanding of the clinical and professional perspectives that is necessary at executive level. Largely, nurse executives are focused on leading nursing practice, culture and development, empowering their teams to operationalise their vision (Cameron and Masterson 2000; Schoville et al. 2022).

Two major factors have influenced the evolution of nurse executive roles-changes to the healthcare environment and regulation. Firstly, as healthcare has become more focused on system-wide requirements, population health and information technology, nurse executives have become central as clinical and system leaders of integrated care (Shortell et al. 1993; Handy 1995; Fedoruk and Pincombe 2000). Secondly, as highlighted by Kerfoot (2001), the evolution of the nurse executive role has often occurred, in many countries where professional standards for nurse executives are explicit and expected. Typically, nurse executives further their knowledge by obtaining graduate education outside nursing management, either in leadership, business, or public administration through attaining a masters or doctoral degree (Barry and Winter 2015).

The United States has been to the forefront in the early establishment and development of the nurse executive role. Wagner et al. (1988) highlight that since the 1970′s, momentum and debate grew across U.S academic institutions on how best to prepare and educate nursing administrators. The American Nurses Credentialing Center (ANCC) Magnet Recognition Program (American Nurses Association 2015) has greatly strengthened nurse executive leadership capacity in United States since its foundation. The model has promoted visible, empowered and accessible nurse executives who encourage and include nurses in decision-making (Upenieks 2003; Stimpfel et al. 2014).

The position of chief nurse was established in the United Kingdom during the 1990s following the implementation of the National Health Service and Community Care Act 1990. One significant study in the United Kingdom, by Cameron and Masterson (2000) who interviewed nurse executive directors, highlighted that many experienced pressure in managing competing demands and there was a lack of dedicated funding for role development. Cameron and Masterson (2000) pointed to nurse executives been dependent on formal power and authority in the United Kingdom that was not obvious in American studies. Filkins (2003) explored the contributions of nurse directors across Germany, Finland, Belgium, Slovenia, Croatia, the Netherlands, France, and the UK, where findings identified challenges in two main areas: the gap between two strong professions—medicine and nursing, and issues relating to education and preparation for the role. More recently, the COVID-19 global pandemic has highlighted that nurse executives have been key leaders, mobilisers and decisions-makers during the crisis; playing a central role in service planning and delivery (Beckman 2020; Daly et al. 2020).

## 2 Background

Despite the growing importance of leadership in healthcare, little is known about how nurse leaders adapt or transition into executive roles. A preliminary search of the literature yielded studies mainly relaying nurse executives experience in the United States and a limited number of studies in the United Kingdom and Europe. These countries have established nurse executive positions at different points, with varying titles, standards, qualifications and education requirements. However, the trajectory of the role has typically emerged from within hospitals, expanding to include the leadership of multiple hospital units, and more significantly, wider healthcare systems (Hafeman 2015). Typically, nursing research has focused on the impact of clinical leadership as a mechanism to improve care outcomes (Antrobus and Kitson 1999; Scully 2015; Labrague 2020). A host of studies have also demonstrated how nursing leadership practices have had a positive impact on indicators such as workforce retention, job satisfaction and well-being (Cummings et al. 2018; Cummings et al. 2021; Specchia et al. 2021). However, the field of executive nursing leadership remains under researched, with little understanding of the complexity of role transitioning for nurse executive leaders. This study aimed to deepen the understanding of how nurse executive leaders navigate the intricate process of role transitioning, providing insights into their experiences and challenges. The study was completed as part of PhD supervised research.

## 3 Methods

### 3.1 Study design

Glaser’s classic grounded theory was chosen as the methodology for this study due to its unique strengths in capturing the complexities of social processes and human experiences (Glaser and Strauss 1968). Glaser (1978) identifies grounded theory as a method that develops theories through systematically gathering and analysing of social research data. Grounded theory achieves this by examining multiple perspectives of the same situation while simultaneously collecting and coding data (Glaser 2003). This approach helps correct bias and enhances objectivity (Thornberg 2012). The method enables the discovery of new meaning and can provide an explanation for what is actually happening in life at a particular time (McCallin 2003).

### 3.2 Study setting

Study participants were nurse executives who were working or had previously held leadership positions either in a permanent or acting capacity for a minimum of six months or more. Initial data was collected from participants recruited across seven hospital networks in the Republic of Ireland and codes were generated from a reflective diary from conversations with six international nurse executives. The study excluded executives from acute hospital settings in other disciplines, such as medicine, human resources and finance.

### 3.3 Recruitment of participants

Purposeful and theoretical sampling strategies were combined to gain information-rich data relating to the area of interest. Initially, purposeful sampling was utilised to identify nurse executives who held accountability for groups of hospitals in the Republic of Ireland. These hospital groups were newly formed, and the nurse executives were tasked with both strategic and clinical leadership roles. Nurse executives were chiefly responsible for guiding nursing practice within their organisations. This encompassed establishing a vision for nursing care, fostering a positive nursing culture, and ensuring that nursing staff are empowered to provide high-quality care. There was potential to recruit up to sixteen participants for this study.

Participants were approached between January 2020 and September 2022. Emails were sent to research participants who met the inclusion criteria of the study. A gate keeper was utilised to access a theoretical sample for the study. Chief Nursing Officers across six countries, England, Scotland, Northern Ireland, New Zealand, Dubai and Australia were contacted following assistance from the Chief Nurse Office, Department of Health, Ireland. The researcher had discussions with six Chief Nursing Officers who volunteered to discuss their role.

### 3.4 Sample and sampling

Participants were twelve nurse executives working in Ireland as described in Table 1, with additional reflective diary notes gathered from conversations with six international nurse executives. Theoretical sampling was applied through the utilisation of a reflective diary. Notes were taken on insights and reflections of the experiences of six international nurse executives and compared to those in the Republic of Ireland. This process helped in delimiting data and supported refining of categories and concepts.

**Table 1:** Key characteristics of participants.

ID	**Age Range**	**Gender**	**Ethnicity**	**Nationality**	**Total years’ experience in the Health Service.**	**Total years’** **experience in a non-nursing management role.**	**Highest** **Academic Qualifications**
1	50–60	Male	White	Irish	31	0	Masters
2	40–50	Female	White	Irish	28	0	Degree
3	50–60	Female	White	Irish	30	2 (Project Manager)	Masters
4	40–50	Male	White	Irish	29	0	Masters
5	50–60	Female	White	Irish	27	0	Higher Diploma
6	50–60	Female	White	Irish	22	3 (General Manager)	Masters
7	40–50	Female	White	Irish	23	0	Masters
8	40–50	Female	White	Irish	25	4 (General Manager)	Masters
9	40–50	Female	White	Irish	20	0	Masters
10	40–50	Female	White	Irish	25	0	Masters
11	40–50	Female	White	Irish	21	0	Masters
12	50–60	Female	White	Irish	35	0	Higher Diploma

### 3.5 Data collection

At the beginning of the study, one self-interview was conducted specifically to help the researcher identify their own bias and assumptions about the phenomenon being studied (Bolam et al. 2003; Ramalho et al. 2015). Glaser (1978) identifies that the analyst who is completely open-minded is frequently more open to what emerges than those who have preconceived perceptions. Interviews were conducted to explore nurse executive’s experiences and main concerns. Glaser (2001) highlights that interview guides can obstruct the process of grounded theory generation; however, prompt questions were developed if required (Supplementary material S1).

Of the twelve participants, seven were interviewed in person, while five were interviewed using the secure online platform Microsoft Teams. This approach was adopted to comply with social distancing guidelines, as data collection occurred during the first wave of the COVID-19 pandemic in the Republic of Ireland. Both face to face and online interviews were unstructured, to enable the interviews to be non-directional, encouraging participants to speak openly and frankly. Interviews were audio recorded, and transcripts generated for coding.

Reflective notes were collated in a diary from conversations with six international nurse executives, comparing the experience of those in the Republic of Ireland. The researchers reflective diary was later coded. Glaser (1978) advocates that whatever is going on in the research field is data, whether it is interviews, observations, reflections or documents (Glaser and Holton 2004).

### 3.6 Data analysis

Grounded theory recommends that the researcher simultaneously collects, codes, and analyses the data from the beginning of the study; hence, data analysis commenced after the first interview. Coding, constant comparison analysis, memo-writing, theoretical sorting and diagramming were applied throughout the data analysis phase, to reconcile a main concern and to raise the conceptual level of the data until theoretical saturation and integration were achieved.

All interviews were conducted in English and transcribed afterwards. The transcripts were checked for errors and inaccuracies by listening again to the recordings. Open coding began after the first interview. During open coding, each interview transcript was analysed line-by-line, and identified incidents were systematically compared to previous codes in a separate analysis document to track building a hierarchy of codes, sub concepts and concepts (Supplementary material S3).

During selective coding, the most frequently repeated codes were synthesised and collapsed, when the same behavioural or social pattern was repeated. Glaser (1978) refers to selective coding as a process where the analyst limits their coding to only those variables that interact with the core variable and this becomes the guide for data collection and theoretical sampling. The interviews became focused on exploring and gaining insight into how nurse executives managed to integrate in their new role.

Data analysis moved to theoretical coding and the relationships between variables and categories were explored, which triggered conceptual mapping. As Gibson and Hartman (2013) identify, a point comes in data analysis when the researcher wants to understand more about the relationship between concepts and theoretical coding. Memos, a vital component of classic grounded theory methodology, were used to record thoughts and interpretations of the data in a memo bank. This process supported the conceptualisation and visualisation of the emerging theory. O’Brien et al. (2019) audit trail template for code generation (Supplementary material S4) was utilised in theory generation.

Glaser et al. (1968) recommends that a theory’s transferability can be further developed by exploring similarities and differences in the data across group and geographical locations. Therefore, the researcher had conversations with six nurse executives from the following countries: England, Scotland, Northern Ireland, New Zealand, Dubai and Australia. The researchers’ diary reflections and memos (Supplementary material S2) were coded and allowed the dependencies and relationships between concepts to be further explored.

Jasper (2005) highlights that reflective journals are an important source of secondary data, providing researcher commentary of their interactions with their data while applying analytical processes. Memos assisted in elevating the conceptual nature of the data, identifying social patterns between concepts. The theoretical coding families (Glaser 1978) utilised in this study are illustrated in Supplementary material S4. Theoretical codes patterned out, producing an integrated grounded theory (Glaser 1998).

### 3.7 Ethical considerations

The study included multiple sites across the acute hospital sector in the Republic of Ireland. Ethical approval was sought and the required authorisation from seven Research Ethics Committees was granted (Supplementary material S5). The inclusion of overseas participants was approved as part of an extended ethical review during the study, where the ethical application accounted for multiple jurisdictions and indicated a commitment to adhere to both the home institution's ethical guidelines and the regulations of the countries where participants are located. There were no major risks identified with this study however, it was acknowledged that research participants may experience feelings of discomfort having to relay negative or challenging experiences during interviews. The researcher was deeply aware that participants agreed to contribute to the study, at a time of extreme pressure in the health service during the COVID 19 pandemic. It was explained that if the interview process became difficult, participants could stop at any time, however this was not required. Research participants were provided with a study information sheet before signing the consent form. All participants provided written informed consent, and pseudonymity was used to protect the identity of research participants, where all transcript data was absent of time, person and place.

## 4 Results

Moving into a new role can be a multifaceted and a varying process for nurse leaders, exposing vulnerabilities and challenges, particularly transitioning to an executive role*.* The key characteristic linking the selected research participants in this study was their experiences of a new executive nursing role. Participants had different role and demographic characteristics such as age, gender, ethnicity and administrative experiences, which were reported utilising a survey (Table 1). Only three participants had experience in non-nursing administrative roles in either project or general management. Academic attainment of the participants ranged from postgraduate diploma to master’s degree qualifications. Of the twelve participants recruited, ten were promoted to new nurse executive positions from within their organisations and two were recruited from outside the organisation in which they took up their new role.

**Fig. 1:**
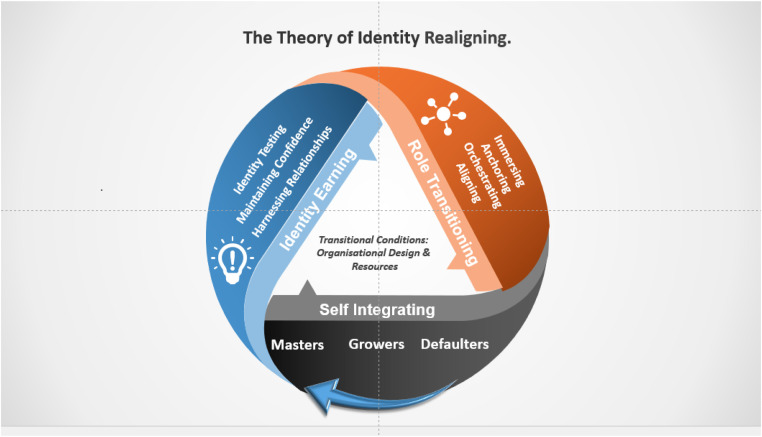
The theory of identity realigning.

The study generated a grounded theory of Identity Realigning, relative to the cultural context of nursing management in the Republic of Ireland. Identity Realigning is a social process which describes how nurse executives realign their identity within a new socio-professional group (Fig. 1). Nurse executives main concern was with “fitting in” (P11). Vignettes and codes are utilised in this paper to detail narratives that encapsulate the personal experiences of participants and are aligned to the four phases identified in the study. In this study, this abstraction is necessary to protect the anonymity of nurse executives in a small county like the Republic of Ireland. 'P' followed by a number (e.g., P3) refers to specific participants and ‘FN' followed by a number (e.g., FN1) refers to field notes.

Nurse executives experience the following four phases:

1. The identity earning phase is a process of generating a new identity relative to one’s new socio professional group and/or surroundings and “letting go” (P4, P7, P8, FN1) of a previous identity. This consists of sub-core categories of identity testing, harnessing relationships and maintaining confidence.
2. The role transitioning phase is a psychological process of moving from one role to another while experiencing a series of different stages: Immersing, Anchoring, Orchestrating and Aligning.
3. The self-integrating phase is an outcome process, which illustrates the degree of assimilation for the individual in a new role and socio-professional group. A typology of self-integration exists where participants demonstrate a preference for being Defaulters (not integrating), Growers (partial integrating) or Masters (integrating).
4. The theory acknowledges two key transitional conditions that impact Identity Realigning: organisational design of the work environment and nurse executives’ access to resources.

### 4.1 Identity earning

**Fig. 2:**
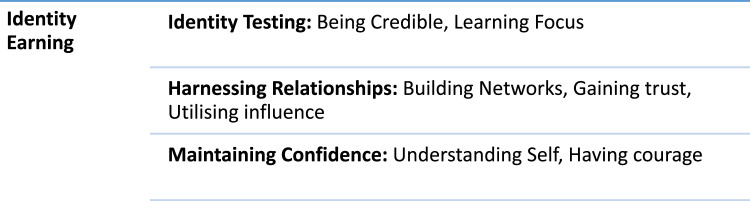
Sub-core categories and properties of identity earning.

Identity earning is the first phase of the theory of Identity Realigning. It is a cyclical process where nurse executives generate a new identity relative to their new socio professional group and “let go” (P4, P7, P8, FN1) of previous identities. The sub-core categories and properties of identity earning are illustrated in (Fig. 2).

Nurse executives earn their identity when they optimise capabilities such as identity testing, maintaining confidence, and harnessing relationships. In this phase, nurse executives begin to test and share their personalities, developing a sense of personal agency, which solidifies and deepens identity. However, when nurse executives struggle with capabilities such as identity testing, maintaining confidence and harnessing relationship, identity earning capacity can be compromised. Thus, nurse executives can have a poor sense of identity and feel disconnected from their surroundings, leading to frustration and self-criticism.

#### 4.1.1 Identity testing

Identity testing is an inward social process where nurse executives experiment with personal identity coming from a desire to achieve congruence within a new socio-professional group. Nurse executives describe

…. trying to establish themselves within the executive management team and the difference there is in this dynamic compared with previous roles (P3, P4, P8, P10, P11).

Through reflexivity, nurse executives often consider the values and behaviours that shape their identity at executive level. Nurse executives are required to fulfil a leadership role, which in turn characterises their identity. Being credible and having a learning focus are essential properties of identity testing.

Nurse executives consider their own determination of credibility. What remains consistent in the findings of this study, is the desire for participants to be perceived as credible by executive peers. Nurse executives are focused on earning trust and confidence and need to be in a position of “being believed” (P10, P12). They strive to align their identity, by being “comfortable in my skin” (FN1, FN4, FN5). Nurse executives who possess a learning focus, can strengthen identity formation quickly. Nurse executives articulated a consensus, that the role

…. provided a steep learning curve, from learning on the job, to acquiring further formal academic education and gaining guidance through conversations with peers (P3, P8, P12, F2 F4, F6).

#### 4.1.2 Harnessing relationships

Harnessing relationships is an outward social process that occurs when nurse executives focus on building networks, gaining trust, and utilising influence generated by new networks. The focus for nurse executives is not on self. Harnessing relationships becomes a mechanism to fuse social connections with people, peer groups, therefore “becoming known” (P3, P4, P7) to others. For nurse executives in early role transition, building networks act as a vehicle to gain knowledge, professional support and advice. Nurse executives highlight

*….* the need for more opportunities to connect with other executive colleagues experiencing role transitioning to gain support… (P3, P4, P7, P9).

Fundamental to harnessing relationships for nurse executives is the motivation to gain trust. Gaining trust is developed by establishing loyalty and consistency in relationships relative to the new socio-professional group. Nurse executives realise and understand the currency and agency this brings, informing professional nursing practice issues.

#### 4.1.3 Maintaining confidence

Nurse executives require confidence to develop a new identity. Maintaining confidence is built over time and certain personal events or junctures can improve or lessen capacity for confidence for nurse executives at any given time. Nurse executives who possess a consistent approach to maintaining confidence possessed a greater likelihood to move forward with opportunities to profile their new identity and part with previous identities. When confidence is low, nurse executives are challenged in reconciling their identity. Nurse executives can feel judged by others depending on their capacity for decisiveness.

Maintaining confidence at nurse executive level requires courage, to manage adversity and challenges. Courageous nurse executives possess greater leadership capacity, which has a positive impact on their ability to earn a new identity. In contrast, to those who lack courage, their identity earning capacity is reduced within their socio-professional group, leading to stress and anxiety.

### 4.2 Role transitioning

Role transitioning is the second phase of the theory of Identity Realigning. Nurse executives in this phase experience a psychological process of moving from one role to another. They engage in four stages which are: Immersing, Anchoring, Orchestrating and Aligning. Nurse executives highlight that

…. getting to know the role, the organisation and what was expected, was challenging, trying to feel your way as a new Chief Nurse, there is often no template. It required defining new governance structures, what would it look like and how will it work. It was all relatively new, trying to find one’s feet. (P2, P6, P9, P10, P11).

For nurse executives, immersing and anchoring stages signify disengaging with an old role, while orchestrating and aligning stages signify engaging towards a new role and socio-professional group. Nurse executives move through all or some of the role-transitioning stages at their own pace. There is potential to experience stages one at a time or concurrently, depending on the individual. Nurse executives who apply a level of commitment to role transitioning will yield a greater degree of self-integration in a new role. Nurse executives who are challenged or do not feel they fit in at executive level, may not align or integrate, defaulting to previous identities.

#### 4.2.1 Immersing

This is the first stage of role transitioning where nurse executives become completely absorbed in their new socio-professional group. This stage occurs when an individual moves for the first time into a new nurse executive role and the focus is inward to self. The goal for nurse executives at this stage is “getting to grips” with the new role (P2, P4, P5, P10, FN 4). Nurse executives who are immersing**,** observe and “soak up” (P5, P10, P11) their new socio-professional world. The action of immersing is driven by a need to preform and achieve. Hence, nurse executives engage in the following strategies: self-analysing, intentional doing, and sense making. These behaviours occur simultaneously, when commencing a new role.

Immersing triggers nurse executives into a process of self-analyse and reflection. Nurse executives critique their actions, attitudes, and behaviours, reflecting on how they are viewed and perceived by others. There is a level of urgency and action associated with immersing, where there is a clear focus on “getting things done” (P4, P5, P12, FN2, FN 5) by intentional doing and sense making. Sense making is a strategy where nurse executives’ fact-find or gain an understanding of information needed to perform. Nurse executives describe

…. just a case of picking up the mantle, go-get on and do it, sometimes going blindly in, often hitting the ground running, as nurses we do the doing and everybody else does the thinking. (P,3, P5, P8, FN4, F6).

#### 4.2.2 Anchoring

This is the second stage of role transitioning where individuals acknowledge their new role within a new socio-professional group. Anchoring occurs after immersing, as nurse executives firmly recognise their new environment, developing strategies to manage within their new role. The focus of anchoring is reconciling and improvising.

Anchoring for nurse executives requires reconciling with previous and current role identities and improvising their way forward. Reconciling occurs when nurse executives accept the reality of their responsibilities and accountability for care. Nurse executives are required to improvise during the transition phase, where they want to deliver the desired response/or intervention that reconciles with their new identity. When nurse executives improvise during role transitioning, it requires agility, critical thinking, collaboration and communication skills. The anchoring stage provides nurse executives time to focus on “bedding down” (P3, P10) and time to adjust. Nurse executives describe

……the significant value at being at the table…nurse executives felt very fortunate to have strong peer networks (P3, P6, P10, P11).

#### 4.2.3 Orchestrating

This is the third stage of role transitioning, where nurse executives are engaging in activities, which build identity and have an intention or desired agenda. Nurse executives can adopt two strategies “playing the game” (P5, FN4, FN6) and building profile. Orchestrating is a deliberate action and efforts are focused on

…. building alliances around clinical management, scope and responsibility, looking at collaboration and partnership all the time, in tandem, trying to look at how to develop services together (P2, P3, P7).

“Playing the game” (P5, FN4, FN6) is a strategy where nurse executives in a new role, operate and perform by what is expected in their new socio-professional group. It requires individuals to be politically active. Nurse executives highlight

… managing the dynamics of the executive team is important……it’s all political and there is a requirement to be politically aware and savvy (P5, P8, FN 6).

Concurrently, nurse executives engage in building their profile, acknowledging that an important component of this is consistency. Nurse executives who demonstrate consistency in their actions, enhance their credibility. When building profile is fully leveraged, there is an increase in nurse executives’ visibility and engagement. Nurse executives advocate

… hold the line, hold a position…… know what you represent, deal with it effectively. . it is not an ego thing (P1, F2, FN6).

#### 4.2.4 Aligning

This is the last stage of role transitioning where nurse executives develop their leadership identity. The focus of aligning is outward within the socio-professional group, where nurse executives are future-orientated. Aligning consists of the properties of “wising up” (FN2, FN5) and holding position.

Nurse executives experience “wising up” (FN2, FN5) where they discern and manage the dynamics of their new socio-professional group. It is action orientated and nurse executives are usually comfortable and astute in managing their new environment. While nurse executives hold position by exerting personal and positional power. This can be exhibited through claiming values, demonstrating excellence in leadership style and behaviour. Nurse executives highlight the importance of

…. Seeing and hearing everything, knowing what is said carries… bringing critical issues to the table (FN3, P1, FN2).

Nurse executives tend to strive to build a new identity that is credible and authentic. The goal of aligning is to ‘fit in’, however for some nurse executives if identity earning capacity is low, they may not succeed in aligning within their new socio-professional group.

### 4.3 Self-integrating

**Fig. 3:**
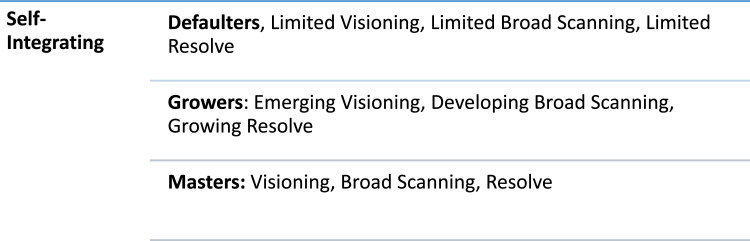
Sub-core categories and properties of self-integrating.

Self-integrating is the third and final stage of the theory of Identity Realigning. It is an outcome process and explains the degree of assimilation for nurse executives in a new role and socio-professional group. Nurse executives are required to bring self, personal experiences and learning to gain maximum integration at executive level. A typology of self-integration exists where nurse executives demonstrate a preference for being Defaulters (not integrating), Growers (partial integrating) or Masters (integrating). The level of self-integrating ranges from not integrating to integrating at executive level. A summary of self-integrating sub-core categories and properties are illustrated in (Fig. 3).

#### 4.3.1 Defaulters

These are nurse executives who do not integrate and tend to hold onto previous role identities, within their new socio-professional group. Hence, their previous role identity hinders integrating at executive level. Defaulters demonstrate a difficulty in articulating a vision and the messaging on direction can be uncertain. Equally, broad scanning capacity is restricted, as their lens and capacity to work across the organisation can be weakened, as they may have had limited experience with executive portfolios. Nurse executives emphasise

…having to upskill, the old toolbox is not sufficient, needing to broaden business lens and focus (FN1, FN3, FN5).

Resolve can be challenging for defaulters as role transitioning to an executive position is daunting and often requires individuals to re assess their capabilities and acquire new competencies. These nurse executives are less likely to hold resolve and can be persuaded away from goals and objectives, becoming disconnected and isolated within their socio-professional group.

#### 4.3.2 Growers

These are nurse executives who partially self-integrate, learning to leave behind previous role identities and adopt to new emerging ones. Nurse executives participate in learning and reflexivity as growers throughout the role transitioning process, which enhances identity earning and connection within their new socio-professional group. There is a shift from previous role identity to developing a new identity. Growers adapt behaviour based on their new socio-professional group. Nurse executives describe the process as

…trying to fit in, lots of learning on the job……it has taken some time to grow into the role (FN5, FN1)

Growers emerge and partially integrate at executive level by clarifying future vision and initiating direction. They are developing broad scanning ability through “connecting trends” (FN1, FN2, FN4) by accessing and formulating information across the organisation. Growers learn to develop resolve by sourcing high support from executive colleagues, peer networks and family.

#### 4.3.3 Masters

These are nurse executives who have integrated at executive level, assimilating within their socio-professional group, gaining a level of mastery. These nurse executives possess comprehensive knowledge and demonstrate a strong identity through visioning, broad scanning and resolve. Individuals acknowledge and adapt to the executive environment, benefiting professional nursing agendas. Masters engage effectively in the political process, bringing health care professionals and systems together. As nurse executives point to

….the need to build good relationships with executive teams’ members, holding position, being comfortable with one’s identity can be extremely important (FN 4, FN2, FN6).

Masters have clarity of vision and can set direction. They possess broad scanning ability, knowing how to access and use information to make decisions across the organisation. They have a strong capacity to hold resolve, demonstrating understanding and resilience to manage complex healthcare issues.

### 4.4 Transitional conditions

The theory of Identity Realigning recognises two important transitional conditional understandings, which influence Identity Realigning: organisational design of the work environment and a nurse executives’ access to resources. The type of organisational design experienced by nurse executives impacts the level of success and engagement in Identity Realigning. A typology of organisational design exists, where nurse executives can experience organisational designs as either a vacuum, emergent or designed in nature. Nurse executives tend to access three different types of resources for support: other executive team members, peer networks and family.

## 5 Discussion

The theory of Identity Realigning provides a framework for understanding the complexities faced by nurse executives during role transitioning, particularly emphasising the unique mechanism of identity earning. Although it has emerged in an Irish context, it holds relevance to international discussions regarding professional socialisation of new nurse executives globally. The study findings were revealed by adhering to the principles of classic grounded theory methodology, employing systematic coding, constant comparison, memo-writing, and theoretical sampling to enhance credibility.

The study recognises role transitioning to be complex and challenging for new nurse executives; it heightens an individual’s focus to adapt to new surroundings. Nicholson (1984) theory of transitioning identifies the initial period of moving into a new role as an adjustment. According to Slotter and Emery (2017) key transitions in people’s lives often interrupt people’s understanding of who they are. Changes that occur during job or career transitions can have a particularly noteworthy impact on an individual (Chick and Meleis 1986). The theory of Identity Realigning identifies that nurse executives often seek learning opportunities to adapt at executive management level. Nurse executives require tailored support to navigate role transitions effectively, to include an understanding of the importance of identity realignment, self-integration, and the development of associated competencies.

The unique concept of identity earning highlights the importance of individuals actively building credibility and trust within their new professional environments. This aspect complements prevailing theories of social identity, such as Tajfel's Social Identity Theory (Tajfel 1979) which emphasises group belonging but does not account for how individuals navigate the complexities of their new roles (Dinmohammadi et al. 2013). The theory of Identity Realigning illustrates that establishing a new identity requires active engagement and significant self-reflection, particularly when confronted with challenges during role transitions. Salisu et al. (2019) indicates that proactive approaches to identity formation can enhance leadership potential, aligning with the assertion that courageous actions can improve one's capacity for effective leadership.

The theory of Identity Realigning articulates three critical stages: identity earning, role transitioning, and self-integrating. This progression captures the evolving nature of professional identity, allowing for a deeper exploration of the psychological and social complexities experienced during transitions. The theory enhances Ashforth’s Role Transition Theories (Ashforth 2000) by providing a detailed view of the psychological and social challenges faced during transitions. The theory of Identity Realigning highlights the importance of self-evaluation in professional life and the opportunity for individuals to test their identities during formative periods. Preparation for executive roles should begin early in a nurse's career. Opportunities such as secondments or associate roles need to be promoted to allow aspiring nurse executives gain relevant experience and familiarity with executive responsibilities.

The unique typology of self-integration, classified into Defaulters, Growers, and Masters, offers insights into how nurse executives navigate their transitions. Understanding these categories provides organisations with guidance on fostering supportive environments that enhance nurse executives' self-efficacy and confidence. Studies support that quality work environments can significantly improve the socialisation processes necessary for successful role adaptation (Rakkarn et al. 2023).

Identity challenges can be exacerbated by the structural and social dynamics present in healthcare settings. Scholars like Rosenberg (1989) point out that individuals are profoundly shaped by their social interactions within their professional contexts, and that social structures provide both the parameters and opportunities for individual’s behaviour (Burke and Stets 2022). The theory of Identity Realigning reflects these complexities, emphasising that nurse executives are both shaped by and shape their environments. Recognising the reciprocal nature of identity construction supports the need for a robust theoretical framework that encompasses the nuances of identity negotiation (Croft et al., 2014). Bespoke developmental opportunities can provide individuals with support in managing their learning requirements effectively. Interventions focused on contextualised leadership strategies and identity negotiation can enhance leaders' ability to navigate complex social dynamics, fostering inclusive environments.

Stryker and Serpe (1982) also highlight that new role transitions can activate feelings of vulnerability. Maintaining confidence can be challenging and requires nurse executives to have a sense of self and courage. Abrams and Hogg (1988) highlight that there are two important processes that take place during social identification, namely self-categorisation and social comparison. Similar, to the theory of Identity Realigning, individuals experience high levels of self-reflection, due to comparing themselves to others. This can sometimes hinder effective transitioning in a leadership role. Alternatively there is evidence that social comparison can sometimes boost motivation and confidence (Guillén et al. 2015). Critically, Guillén et al. (2015) signal how having access to role models in an organisation can boost nurse executive confidence. Healthcare organisations need to continually work to actively assure the presence of onboarding role models and mentoring opportunities for nurse executives. Bennett (2021) also highlights that executive leaders tend to seek learning mechanisms such as coaching for building confidence, as it drives understanding of what is going on and why, by examining the situation in a safe space. Such support structures can help nurse executives gain the confidence needed to establish their identities in a new context (Ryan et al. 2024).

Identity Realigning highlights how harnessing relationships and gaining trust can support nurse executive transition successfully. Many writers in leadership literature support the importance of developing relationships as an essential contributor to performance (Tieranta and Yliniemi 2021; Ferrucci and Fitzpatrick 2024). The necessity for nurse executives to leverage connections, plays a central role in professional success (Koskiniemi et al. 2019). Hence, the provision of induction programmes for new nurse executives can be beneficial in facilitating networking opportunities during transition.

The existing body of literature suggests that as individuals ascend to higher roles within an organisation, they often acquire new competencies while relinquishing outdated ones (McGill et al. 2019). Nurse leaders, when promoted to executive positions, frequently face challenges in adapting to the necessary executive skill set. Research conducted by Wetsel et al. (2019) outlined the perceptions of interim Chief Nursing Officers, revealing that the acquisition of business skills is deemed the highest priority. Similarly, Nguyen et al. (2019) identified a recommendation for physician leaders to cultivate business acumen to navigate the corporate dimensions of healthcare effectively. There is a significant consensus in the literature that proficiency in business acumen, along with interventions such as coaching, can enhance skills, foster career advancement, promote emotional intelligence, and improve overall performance (Shanafelt et al. 2015; Loh et al. 2016; Kragt and Day 2020). However, there has been insufficient emphasis on the systematic cultivation of clinical leadership and management skills in both undergraduate and postgraduate nursing programmes (Spurgeon and Clark 2017). The integration of subjects such as business, informatics, service planning, and systems leadership into higher education curricula could yield substantial benefits (Wise and Payne 2022). From a policy perspective, to ensure a consistent and supportive approach to the preparation of nurse executives, the establishment of Professional Practice Standards is recommended. These standards would offer guidance on the competencies required for nurse executive leadership in evolving healthcare environments.

Identity Realigning provides an understanding of the journey faced by nurse executives as they transition into leadership roles. By outlining the processes of identity earning and the importance of social interactions, the theory makes a significant contribution to the existing literature on professional socialisation and identity formation. Specifically, it highlights the need for targeted support systems in healthcare that facilitate role transitions and encourage the development of credible and confident nurse leaders.

### 5.1 Strengths and limitations of the study

The theory of Identity Realigning details the new concepts of identity earning, the process of role transitioning, the typology of self-integration and new knowledge relative to transitional conditions i.e., the organisational design and resources. It has potential for transferability as a formal theory to other sectors e.g., industry and education. A limitation of the study was the research sample, which was predominately female. The research sample was also limited to Ireland, which may affect the transferability of the findings to countries with differing socio-cultural and healthcare contexts.

## 6 Conclusions

In conclusion, this classic grounded theory study significantly advances our understanding of the experiences of nurse executives as they navigate the complexities of role transitioning within a new socio-professional group. Participants articulated their experiences of stepping into leadership roles with a sense of urgency and determination, often feeling without adequate preparation or support. This highlights the need for structured support systems and educational programmes that can better equip nurse executives. By understanding the intricacies of identity transformation in nursing leadership, educators and policymakers can develop targeted strategies that foster resilience and adaptability among nurse executives. Healthcare organisations can leverage the findings of this study to inform policy and resource allocation, ensuring that nurse executives are equipped with the necessary tools to succeed.

This study contributes to broader theoretical developments by challenging existing paradigms and introducing new frameworks that can be utilised in future research. Specifically, further research needs to explore, at what point do nurse executives feel integrated as a team member at executive level. There is also a legitimate opportunity for further development of the theory of identity realigning by exploring its relative concepts outside the profession of nursing, applying the theory’s potential with other executive leadership environments such as industry or education.

## Funding

The primary author of this manuscript was a recipient of a Government of Ireland Postgraduate Scholarship and gratefully acknowledges the financial stipend provided by the Irish Research Council for this study.

## CRediT authorship contribution statement

**Cora Lunn:** Writing – review & editing, Writing – original draft, Validation, Resources, Project administration, Methodology, Investigation, Funding acquisition, Formal analysis, Data curation, Conceptualization. **Claire O’ Donnell:** Writing – review & editing, Supervision, Methodology, Conceptualization. **Sarah MacCurtain:** Writing – review & editing, Supervision, Conceptualization. **Alice Coffey:** Writing – review & editing, Supervision, Conceptualization.

## Declaration of competing interest

The authors declare the following financial interests/personal relationships which may be considered as potential competing interests: Cora Lunn reports financial support was provided by Irish Research Council. If there are other authors, they declare that they have no known competing financial interests or personal relationships that could have appeared to influence the work reported in this paper.

## Appendix Supplementary materials

Image, application 1

Image, application 2

Image, application 3

Image, application 4

Image, application 5
