# Supplementary material for: Realigning identity: Nurse executives' experiences within a new socio-professional group – A classic grounded theory study
Source: Int J Nurs Stud Adv. 2025 Jun 14;9:100367. doi: 10.1016/j.ijnsa.2025.100367 (PMC12214279; doi:10.1016/j.ijnsa.2025.100367)
Supplement: Supplementary file 1 [file mmc1.docx]

**S1 Interview Prompt Questions -Guide**

1. Tell me about your experience of your role…?
2. Tell me about the journey so far ... ?
3. Tell me about transitioning into this new role…? How is it going?
4. Tell me about what has stood out for you?
5. Tell me about your responsibilities?
6. What new insights have you gained?
7. Tell me about what has influenced your journey?
8. Tell me about your challenges…?
9. Tell me about your ambitions and goals?
10. Tell me about what supports you?
