# Supplementary material for: Realigning identity: Nurse executives' experiences within a new socio-professional group – A classic grounded theory study
Source: Int J Nurs Stud Adv. 2025 Jun 14;9:100367. doi: 10.1016/j.ijnsa.2025.100367 (PMC12214279; doi:10.1016/j.ijnsa.2025.100367)
Supplement: Supplementary file 2 [file mmc2.docx]

**S2 Memo Writing Extract**

**Emerging Role (Organisational)**

Role is emerging… to what extent is the role designed, is there also an emerging role or evolving role… which is better? Very little predesign of role, participants refer to all the time as having nothing to refer to. Participant spoke about operating on fly, and high volumes of change in the system. So the structural conditions influence the evolution and design of the role.

**Context**

Role and structural condition… this role evolves from government policy and not through the profession. Challenging environment - domino and cascading effect when the nurse executive role emerges. Leading to redesign layers -organisation reacting vs planning mode. There is no planning for the post so what do participants do when they move into post. They tend have to negotiate resources. Something around the onset of the role, the context of which people were working in was austerity (limited resources), but also there was limited staffing. They are navigating the role as it emerged in different stages in different hospitals. The role is still evolving – navigating role emergence.

**Developmental Stages (phased transitioning)**

There is a combination of organisation and individual effort (design and practice) that influence their transition to a new role. The culture drives operational agenda, but the role expectation is to be strategic. There is a tension here. The focus is on getting to grips and getting thing done- hitting the ground running.

**Operating (Emerging) on the fly**

Individual seeks to live the designed role but is designed role incomplete? Designing on the fly. This seems to be an adaptive strategy.

**Resourceful Transitioning**

There is alot effort into focusing on establishing the role themselves, absence or conversation or dialogue on induction or transitioning support. They have had to find their own way/ As part of this emergence, it is that what nurses do we get on and do it- adaptive nature – a resilience to manage ‘*hit the ground running’*

**Stepping up**

Responsible for delivering nursing leadership versus now the new role of executive responsibilities – developing identity at executive level. Balancing demands, managing a hospital group plus new executive responsibilities ongoing. Role leading to change in governance lines- role resulting in cascading changes. Interviewee reflects a lot on whether these structures have worked.
