# Supplementary material for: Realigning identity: Nurse executives' experiences within a new socio-professional group – A classic grounded theory study
Source: Int J Nurs Stud Adv. 2025 Jun 14;9:100367. doi: 10.1016/j.ijnsa.2025.100367 (PMC12214279; doi:10.1016/j.ijnsa.2025.100367)
Supplement: Supplementary file 3 [file mmc3.docx]

# **S3 Open Coding Extract**

| **Exemplar Quote** | **Open Code** |
| --- | --- |
| When I was in the role, it was in 2019. It was a new role that had been established late. And as a result, it was quite new in its development and quite new in as an organisation. We had new hospital groups. | Establishing role  Emerging role  New to organisation  Emerging hospital groups |
|  |  |
| We had really only established the group working in 2019. And so the role as the chief director of nursing and midwifery was still in the evolving stage of where the organisation was kind of setting up. So I got no kind of storming phase in all the initial organisational changes. The directors of nursing were the direct reports into the role, were established and they were still working through setting up the group structure of the directorate structure. And they were still very much focused on the operational piece. | Evolving role within new structures  Emerging organisational change  No storming phase  Organisational change  Emerging organisational change  Operating |
|  |  |
| So the experience in the role, was that it was a extremely busy role. It was an extremely challenging role and it was very diverse. You obviously had your nursing leadership pillar that you had to deliver against, but also had your corporate pillar and your board be an executive member of the board that you had to deliver to. So while your brief, was really broad within nursing and encompassed six hospitals. And the challenges also were that you were trying to balance that with the demands of the corporate role and the corporate piece as well. | Extremely busy role  Challenging role  Diversing  delivering nursing leadership  broading responsibilities and remit  delivering corporate and executive responsibilities  Balancing demands |
