# Supplementary material for: Realigning identity: Nurse executives' experiences within a new socio-professional group – A classic grounded theory study
Source: Int J Nurs Stud Adv. 2025 Jun 14;9:100367. doi: 10.1016/j.ijnsa.2025.100367 (PMC12214279; doi:10.1016/j.ijnsa.2025.100367)
Supplement: Supplementary file 4 [file mmc4.docx]

**S4** Code Generation

| O’Brien *et al.* (2019) Audit Trail for Code Generation  Audit Trail of a code, invivo codes/open codes. | Getting things done  Taking the bull by the horns  Moving forward  Getting results  Picking up and going  Getting on with it  Rolling up the sleeves  Doing it  Sucking and seeing  Hitting the ground running  Focusing on outputs  Getting the job done  Doing the doing  Getting on  Getting on with things  Focusing on doing  Getting to grips  Picking it up quickly  Doing with actions  Just doing it  Hit the ground running |
| --- | --- |
| Connection to Selective Coding | Conceptualised to *Intentional doing* |
| Connection Category | *Intentional doing* is a property of *Immersing* |
| Connection to Core Concepts | *Immersing* is a sub concept of *Role-Transitioning,* which is a core concept of I*dentity Realigning.* |
| Connection Core Category | *Intentional doing* is a sub concept of *role transitioning,* which is a concept of *Identity Realigning* -the core concept of the study.  *Identity Realigning* i*s the strategy adopted by nurse executives to realign their identity within a new socio-professional system.* |
| Theoretical Codes | The strategy family featured as a main theoretical code to shape this theory as it provided possibilities for the integration of substantive codes, concepts and properties relative to the core concept and core category. |

Theoretical Coding Families

| Theoretical Coding Families | Significance for the theory of Identity Realigning |
| --- | --- |
| 6C’s Family | Includes causes, context, contingencies, consequences, covariance’s, and conditions. |
| Process Family | The process family emerged as the predominant theoretical code, where nurse executives adopt Identity Realigning to manage their main concern “finding my place” (P11).  The concept of identity earning, and role transitioning emerged as social processes where there were stages, steps, shaping or cycling |
| Degree Family | The concept of self-integrating belongs to the degree family, where the focus is on range, limits intensity, extremes, polarity, full, partial etc. |
