# Supplementary material for: Realigning identity: Nurse executives' experiences within a new socio-professional group – A classic grounded theory study
Source: Int J Nurs Stud Adv. 2025 Jun 14;9:100367. doi: 10.1016/j.ijnsa.2025.100367 (PMC12214279; doi:10.1016/j.ijnsa.2025.100367)
Supplement: Supplementary file 5 [file mmc5.docx]

| **Reference Number** | **Research Ethics Committee Site Applications** | **Date Approved** | **Sample Total & Recruitment Phase** |
| --- | --- | --- | --- |
| Ref 112/19 | University of Limerick Hospital Group, Ireland | October 2019 | 5 Participants recruited between January 2020 -December 2020 |
| No reference number stated on letter. Letter date 15/11/2019 | Corporate HSE (HSE Research and Development Team), Dublin Ireland | November 2019 | 1 Participants recruited between January 2020 -December 2020 |
| REF: C.A 2617 | Saolta Hospital Group, Galway Ireland | April 2021 | 1 Participants recruited between September 2021 -December 2021 |
| SKM_227_Col21042209191 | Dublin Midlands Hospital Group (via Tallaght Hospital), Ireland | October 2021 | 2 Participants recruited between January 2022 -September 2022 |
| REC REF: GEN/944/21 | Children’s Health Ireland (CHI via Crumlin Hospital), Dublin, Ireland | AUGUST 2021 | 1 Participants recruited between January 2022 -September 2022 |
| IRBR: 1/378/2267 | Ireland East Hospital Group (via Mater Hospital), Dublin, Ireland | NOVEMBER 2021 | 1 Participants recruited between January 2022 -September 2022 |
| ECM 4 (hh) 11/05/2021 & ECM 3 (jjj) 01/06/2021 | South/Southwest Hospital Group, Cork Ireland | June 2021 | 1 Participants recruited between September 2021 -December 2021 |

**S5 Seven Research Ethics Committees Approval References.**
